# Hidden diversity: DNA metabarcoding reveals hyper-diverse benthic invertebrate communities

**DOI:** 10.1186/s12862-023-02118-w

**Published:** 2023-05-17

**Authors:** Jennifer Erin Gleason, Robert H. Hanner, Karl Cottenie

**Affiliations:** 1 Ottawa Research and Development Centre, Agriculture and Agri-Food Canada, Ottawa, ON Canada; 2 Department of Integrative Biology, University of Guelph, Guelph, ON Canada

**Keywords:** Aquatic invertebrates, Biodiversity, Biomonitoring, Metabarcoding, Stream ecology

## Abstract

**Background:**

Freshwater ecosystems, such as streams, are facing increasing pressures from agricultural land use and recent literature stresses the importance of robust biomonitoring to detect trends in insect decline globally. Aquatic insects and other macroinvertebrates are often used as indicators of ecological condition in freshwater biomonitoring programs; however, these diverse groups can present challenges to morphological identification and coarse-level taxonomic resolution can mask patterns in community composition. Here, we incorporate molecular identification (DNA metabarcoding) into a stream biomonitoring sampling design to explore the diversity and variability of aquatic macroinvertebrate communities at small spatial scales. While individual stream reaches can be very heterogenous, most community ecology studies focus on larger, landscape-level patterns of community composition. A high degree of community variability at the local scale has important implications for both biomonitoring and ecological research, and the incorporation of DNA metabarcoding into local biodiversity assessments will inform future sampling protocols.

**Results:**

We sampled twenty streams in southern Ontario, Canada, for aquatic macroinvertebrates across multiple time points and assessed local community variability by comparing field replicates taken ten meters apart within the same stream. Using bulk-tissue DNA metabarcoding, we revealed that aquatic macroinvertebrate communities are highly diverse at small spatial scales with unprecedented levels of local taxonomic turnover. We detected over 1600 Operational Taxonomic Units (OTUs) from 149 families, and a single insect family, the Chironomidae, contained over one third of the total number of OTUs detected in our study. Benthic communities were largely comprised of rare taxa detected only once per stream despite multiple biological replicates (24–94% rare taxa per site). In addition to numerous rare taxa, our species pool estimates indicated that there was a large proportion of taxa that remained undetected by our sampling regime (14–94% per site). Our sites were located across a gradient of agricultural activity, and while we predicted that increased land use would homogenize benthic communities, this was not supported as within-stream dissimilarity was unrelated to land use. Within-stream dissimilarity estimates were consistently high for all levels of taxonomic resolution (invertebrate families, invertebrate OTUs, chironomid OTUs), indicating stream communities are very dissimilar at small spatial scales.

**Supplementary Information:**

The online version contains supplementary material available at 10.1186/s12862-023-02118-w.

## Background

As the effects of climate change become more severe and we enter a sixth mass extinction event [1], it is now more than ever critical to conserve vulnerable habitats and slow the rate of biodiversity loss. While trends in vertebrate species have been easier to document and traditionally received more attention [e.g., 2–4], the importance of insects and threats towards them have garnered a broader interest in the past decade with the publication of alarming trends in insect decline. For example, Hallman et al. [5] estimated that there has been a 75% decline in flying insect biomass in Germany since the late 1980s, and Sánchez-Bayo and Wyckhuys [6] have predicted that 40% of insect species will be extinct in the next few decades. While there has been debate whether the decline will be as severe as Sánchez-Bayo and Wyckhuys [6] predicted [e.g., 7], it nevertheless remains clear that there is a consistent pattern of insect decline across a broad range of taxonomic groups and habitats in response to climate change and land use [8–10]. Freshwater habitats, such as streams, are particularly threatened by anthropogenic land use and climate change, despite their irreplaceable ecosystem services [11, 12]. The biodiversity of freshwater systems is declining at a faster rate than marine or terrestrial habitats [13], stressing how essential stream biomonitoring and conservation projects are to preserve these ecosystems. Biomonitoring assessments often incorporate aquatic invertebrates, which are key bioindicator taxa and sensitive to habitat disturbances [14]. However, these groups present challenges to traditional morphological identification. For example, many immature larval stages cannot be reliably identified to species-level due to the lack of diagnostic characters which can result in identification errors [15, 16]. Due to these constraints, environmental assessments using morphological identifications often use coarse taxonomic resolution (e.g., family-level) as a surrogate for species-level identification, which could potentially mask species-level turnover within a family or not prove sensitive enough to detect impairment [17]. The limitations in time and financial resources, large volumes of samples, and either coarse taxonomic resolution or narrow taxonomic focus can be impediments to monitor stream systems exposed to complex physical and chemical stressors.

The above challenges, combined with the need for species detection in an ongoing biodiversity crisis, has prompted research programs which suggest that molecular tools can provide a promising future for freshwater biodiversity assessments [18–20]. Over the past decade, there have been major advancements in molecular identification tools (e.g., high-throughput sequencing or metabarcoding; [21, 22]), which have the potential to be incorporated into biomonitoring programs and ecological research. Metabarcoding has huge potential for environmental assessments as high-throughput sequencing (HTS) platforms can efficiently sequence and identify entire samples [22, 23]. In previous studies comparing morphological identification and DNA metabarcoding of invertebrate communities, molecular methods have proven either equally or more effective than traditional approaches at investigating patterns of biodiversity [23–26]. Very speciose taxa can especially benefit from metabarcoding applications, such as the family Chironomidae (non-biting midges, a ubiquitous group of flies with a freshwater larval stage), which occupy most freshwater habitats and are often grouped at family-level resolution in assessments [27].

While metabarcoding provides an avenue for efficient and cost-effective macroinvertebrate identification for biomonitoring programs, the variability of stream habitats can make it challenging to determine whether sampling efforts have been sufficient. Local micro-habitat level conditions, such as riparian vegetation, sediment type, organic matter, and flow regime (e.g., riffle versus pool), are important factors in structuring benthic communities [28, 29]. Since stream microhabitats can vary on small spatial scales, this creates a very patchy system in terms of physical stream attributes and resources, and thus affects community composition of aquatic invertebrates [30]. This spatial extent knowledge gap, combined with the potentially unknown taxonomic diversity of benthic invertebrates, can be uniquely answered by sampling methods based on metabarcoding principles to inform biomonitoring protocols and efficiently record biodiversity in stream systems.

Small-scale variations in stream habitats can be caused by both natural and anthropogenic processes, although loss of microhabitats (e.g., habitat homogeneity) is often associated with adjacent agricultural land use, which results in channelization and reduction of riparian vegetation [31]. Heterogeneous habitats tend to support higher species richness [32, 33], and it is perhaps unsurprising that increasing agricultural land use in the surrounding catchment area can cause taxonomic and functional homogenization in aquatic macroinvertebrates, resulting in more similar communities of ‘tolerant’ taxa [34, 35]. However, the relationship between beta diversity (a measurement of community dissimilarity) and both land use and habitat heterogeneity in stream macroinvertebrates is unclear and often case dependent. In Finland, Heino et al. [36] determined that heterogeneity was not a significant predicator of invertebrate beta diversity in streams using a mix of species and genus-level identifications. Additionally, research using predominately genus-level identifications by Petsch et al. [37] concluded that land use did not cause homogenization of stream invertebrates in boreal (Finland) or subtropical (Brazil) regions. However, contrasting patterns have been detected in New Zealand, where habitat heterogeneity was a strong driver of beta diversity in stream invertebrate communities [38] and in North America (Maryland, USA) where Maloney et al. [39] detected a negative relationship between beta diversity and increased pasture and crop cover. The above studies of stream invertebrate beta diversity patterns are performed at large spatial scales (e.g., comparing beta diversity between major watersheds or geographic regions), and there are few stream studies that explore spatial resolution at small scales (e.g., microhabitat level, but see [36, 40]).

In this study, we used bulk tissue DNA metabarcoding to determine how variable benthic invertebrate communities are at small spatial scales across three time points in a single year. We determined the importance of taxonomic resolution in revealing biodiversity patterns by performing all analyses at both family-level and OTU-level resolution. As chironomid OTUs generally comprise high levels of diversity in freshwater samples, we also repeated all analyses using only OTUs from this family. We assessed whether overall taxonomic richness is linked to land use and calculated within-stream dissimilarity to determine if small-scale changes in community composition are influenced by agricultural activity. We hypothesized that agricultural landscapes homogenize stream communities and cause more uniform benthic communities due the loss of habitat complexity, and therefore predicted that within-stream dissimilarity will decrease (e.g., more homogenous communities) as the percentage of agricultural land use in the catchment increases. We also explored how stream biodiversity estimates change between different levels of taxonomic resolution by calculating rarefaction curves and estimating the total regional species pool, calculating the sampling coverage at each stream site and determining the percentage of a local community made up by rare taxa in order to inform future sampling efforts.

## Methods

### Site selection and stream sampling

**Fig. 1:**
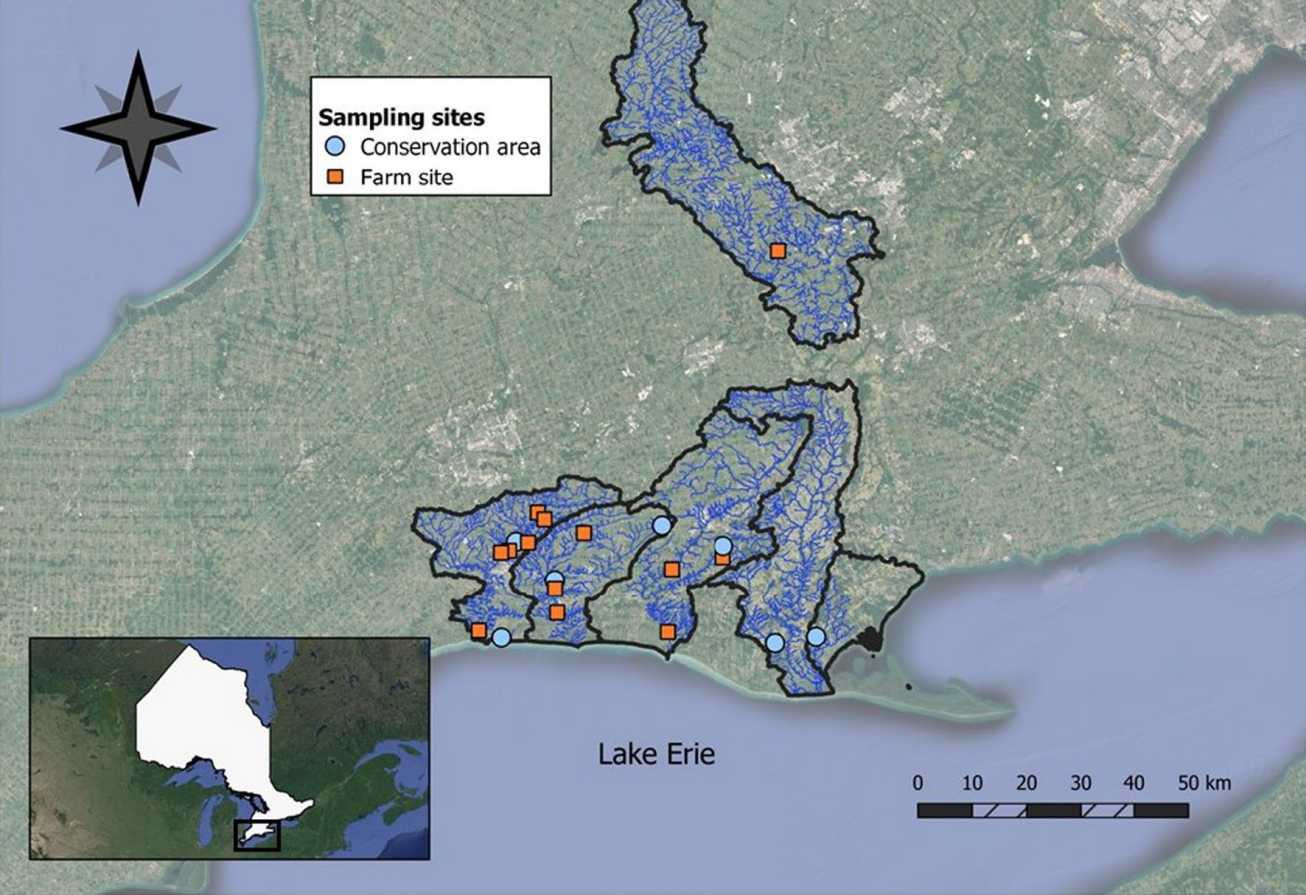
A map of stream sampling sites in conservation areas (blue circles) and privately owned farms (orange squares) in southern Ontario, Canada, along the north shore of Lake Erie. The map was created using QGIS [46]. The black outlines demonstrate quaternary watershed boundaries where we sampled. The inset map shows the province of Ontario in white with our study region outlined in a black rectangle

We collected benthic macroinvertebrates from twenty streams in southern Ontario across three time periods (May, July, and September 2019; Fig. 1). We selected streams on a continuum of surrounding land use, and sites were located either on Conservation Authority property or privately owned land (farm sites), and additionally were required to be wadable and wet for the entire study period. We used the Ontario Flow Assessment Tool (OFAT) [41] to determine stream watershed boundaries in ArcGIS v. 10.6.1 [42] and the Ontario Land Cover Compilation v. 2.0 [43] to determine the percentage of agriculture land use (cropping) surrounding each stream site.

For field collection of aquatic macroinvertebrates, we collected four biological replicates within each stream (i.e., four bulk samples per stream) by selecting four transects that were approximately 10–20 m apart and positioned downstream to upstream to avoid contamination from sampling-related disturbance. We placed transects to include multiple microhabitats if present (e.g., riffles and pools, different substrates) and collected benthic macroinvertebrates and associated habitat information based on the Ontario Benthic Biomonitoring Network (OBBN) [44] and the Ontario Stream Assessment Protocol (OSAP) [45]. Each sample consisted of a 3-minute travelling kick-and-sweep using a 500 μm D-net across the width of the stream. We then transferred the bulk sample to a 500 μm mesh sieve for rinsing and the removal of large debris, before storing in a sample container and preserving in 95% ethanol on site. We kept the invertebrate samples in a chilled cooler until transfer to the lab on the same day, where they were stored in a 4 °C fridge until further processing. All sampling equipment (e.g., nets, sieves, forceps, waders) was cleaned with a 10% bleach solution and rinsed with de-ionized water (DI) between sites. In total, we collected four biological replicates per stream and 80 samples each month, for a total of 240 bulk samples.

### Sample sorting and DNA extraction

Bulk macroinvertebrate samples were rinsed with DI water over a sterilized 500 μm sieve and sorted under a dissection microscope. Benthic macroinvertebrates were removed from sample debris and placed in a sterile 20 mL tube containing 95% ethanol and ten 4 mm diameter steel beads for later homogenization. As many bulk samples were very large, we used a subsampling approach based on equal effort by stopping sorting after 4 h had elapsed. The unsorted portion of sample was placed on a white grid and scanned for 2 min for any rare taxa that had not been encountered during the initial subsampling. After sorting had been completed, excess ethanol was removed by a pipette and samples were air dried for 1 week while covered with a kimwipe. The dry biomass of each sample was recorded, and then samples were homogenized within their sampling tubes using an IKA Tube Mill (IKA, Staufen, Germany) at 4000 rpm for 15 min. Smaller samples were ground in a 2 mL sterile tube with two steel beads using a TissueLyser II (Qiagen, Hilden, Germany) at 30 Hz for 1 min. We subsampled 20 mg (± 1 mg) of ground tissue into a sterile 2 mL tube and used a DNeasy Blood & Tissue Kit (Qiagen, Hilden, Germany) following manufactures’ guidelines for DNA extraction, follow by quantification using a Qubit 3.0 Fluorometer (ThermoFisher Scientific, MA, USA). Several samples contained less than 20 mg of tissue, and the entire sample was used for DNA extraction in place of sub-sampling.

### PCR amplification and library preparation

We used a two-step PCR library preparation protocol to first amplify our target region followed by a second indexing reaction [see 47]. For the first step, we used the Qiagen multiplex PCR kit (Qiagen, Hilden, Germany) as our master mix and selected a primer pair (BF2 + BR2; [48]) that has been successful at amplifying a broad range of invertebrate taxa, including aquatic invertebrates collected from our study region [49, 50]. We selected the mitochondrial cytochrome c oxidase subunit I (CO1) gene as our marker and the BF2 + BR2 primer set targeted a 421 bp region to amplify in our initial PCR reaction. Each reaction consisted of 2.5 µL DNA extract, 12.5 µL of 2x Qiagen master mix, 9 µL of molecular water, and 0.5 µL of each primer (BF2 + BR2, reaction concentration of 0.2 µM) for a total reaction volume of 25 µL. Our thermocycling profile followed Qiagen’s manufacturer’s protocol: a 95 °C initial denaturation for fifteen minutes, followed by 25 cycles of 94 °C for 30 s, 50 °C for 90 s, 72 °C for 60 s, and a final extension at 72 °C for ten minutes and visualized using precast 2.0% agarose e-gels (E-Gel 96 SYBR Safe DNA stain; ThermoFisher Scientific, MA, USA). Each sampling period (e.g., month) consisting of 80 samples were prepared in their own plate, in addition to 6 PCR negative controls, 1 sequencing negative control, and 1 extraction negative control. We included 8 PCR technical replicates per plate to ensure PCR reproducibility and explicitly selected samples of both lower and higher invertebrate abundance. This resulted in three 96-well PCR plates consisting of 240 samples, 24 technical replicates, and 24 negative controls. The resulting PCR products were purified using NucleoMag NGS clean up and size select magnetic beads (Macherey-Nagel, USA) with an 0.8x ratio of beads to PCR product as per Milián-García et al. [51].

A second PCR reaction was prepared using indexing primers to tag samples for library preparation. Here, we prepared a 50 µL PCR reaction using 5 µL of our purified PCR product, 25 µL of 2x Qiagen master mix, 10 µL of molecular water, and 5 µL each of forward and reverse indexing primers (initial concentration 10 µM) based on Illumina’s standard indexing protocol. The thermocycling profile included an initial denaturation at 95 °C for fifteen minutes, 8 cycles of 95 °C for 30 s, 55 °C for 30 s, 72 °C for 30 s, and a final extension of 72 °C for five minutes. After e-gel visualization to confirm amplification, we again purified the PCR products using NucleoMag beads (0.6x ratio, [51]). After a final visualization, we submitted the prepared libraries to the Advanced Analysis Center at the University of Guelph. Each plate was normalized, pooled, and sequenced separately on the Illumina MiSeq platform for a total of three separate runs.

In some cases, samples did not perform well in sequencing and were subsequently filtered out of the dataset based on low sequence read (36 samples filtered out with fewer than 80k sequences). Most of the failed samples came from the same streams and had lower-than-average DNA concentration, and we re-ran these samples following the same protocol as above, but instead increased the template volume to 5 µL in the initial amplification PCR and added an additional 10 cycles to the thermocycling program for the first PCR (CO1 amplification) and submitted a fourth plate for sequencing as above to replace failed samples.

### Bioinformatics pipeline

We used the bioinformatics platform JAMP v. 0.67 (http://github.com/VascoElbrecht/JAMP) to process the raw sequence data. The protocol is listed in detailed in Persaud et al. [50], but in brief this involved paired-end merging of de-multiplexed reads using USEARCH v. 11.0.6668 [52] followed by trimming primer sequences from reads using cutadapt v. 1.15 [53]. We assessed sequence size by filtering out any that were more than ten base pairs longer or shorter than our target (421 bp) and filtered out low-quality sequence with expected errors ≥ 1. We used USEARCH v. 11.0.6668 [52] to cluster quality sequences from all runs into Operational Taxonomic Units (OTUs) using a 97% similarity threshold, and OTUs with less than 0.01% abundance across all samples were filtered out [24, 54]. We matched our OTUs to the Barcode of Life Data System reference sequence library (BOLD) [55] using the Python program BOLDigger [56]. Raw sequences are available on NCBI’s Sequence Read Archive (BioProject ID: PRJNA783201) and our final OTU table with sequence reads per sample and associated taxonomic metadata are available as supplementary information.

### Data quality control

All of our statistical analyses and figures were performed using R version 4.0.3 [57], and all plots were created using the package ggplot2 v.3.3.3 [58]. We used the R package metabaR v. 1.0.0 [59] to assess the quality of our metabarcoding data, including confirming sequencing depth was appropriate and checking for contamination on sequences present in our negative controls (see Additional file 1: Appendix 1 for further details). A sequence was identified as a contaminate if it had a relative abundance that was highest in a negative control (as no other DNA should be present in negative controls, contaminants should be preferentially amplified). A sample would be flagged as failed if more than 10% of its total sequence reads corresponded to an OTU identified as a contaminant. Based on the small number of reads in sequencing controls, we tested multiple filtering thresholds to lower the influence of tag jumps and prevent false positives. The abundance of an OTU in sample was changed to zero if the relative abundance of that OTU was less than 0.001% of the total abundance of that OTU in the entire dataset. We then filtered out samples with low sequence reads (less than 1 SD below average sequence read; 87,344) and assessed the quality of technical replicates for reproducibility based on Bray-Curtis distances within and between samples (e.g., contrasting the dissimilarities in OTU composition). A sample was flagged as a failed if the distance within a sample (e.g., between technical replicates) was greater than the threshold of the intersection value for within and between sample distances. We filtered out any non-target taxa and retained only arthropods, annelids and molluscs which were the three must abundant phyla in terms of both total sequence reads and OTU counts. We did not include nematodes in any further analysis as we did not obtain many sequences or OTUs matching to this group, likely due to a combination of the small size of some species and primer bias. Finally, we filtered out poor-quality taxonomic matches (< 90% match to reference database). After cleaning the data using metabaR, we calculated the total number of sequences and OTUs that were removed from the dataset during this process. See Additional file 1: Appendix 1 for additional details and figures for the above protocol.

### Statistical analysis

To assess the influence of taxonomic resolution on ecological patterns, we analyzed the data first at family-level and OTU-level resolution. We additionally analyzed the chironomid OTUs on their own as this family made up a large portion of the diversity within our dataset. All data were analyzed at three time points in our data set (May, July, September). To determine if taxon richness varied between stream type (e.g., located in a conservation area or on private property) or between sampling months, we used the ‘lmer’ function in the R package lmerTest v. 3.1-3 [60] to perform a mixed effects model to test the significance of site type and sampling month (and the interaction between them) on taxa richness for each identification level. We incorporated stream site as a crossed random effect to account for all streams being repeatedly sampled each month. We used lmerTest to generate *p* values for the fixed effects in the above and all subsequent models by using Satterthwaite’s method to approximate degrees of freedom for all F-tests (see [60]).

To assess how within-stream dissimilarity is influenced by surrounding land use, we calculated the Raup-Crick index between the four biological replicates (transects) within a stream using the ‘raupcrick’ function with 999 simulations in the R package vegan v. 2.5-7 [61] and then took the mean of all pairwise comparisons as the dissimilarity value for that site. Using Raup-Crick as a dissimilarity index is ideal for metabarcoding data as it treats the number of sequence reads per OTU as binary (e.g., presence/absence) as opposed to an abundance value, and it is based on occurrence probabilities in proportion to frequency of a species occurrence and thus should be robust to the influence of rare taxa and large differences in richness between sites. We calculated a mixed effects model for each identification level to test if within site dissimilarity (i.e., the mean of all within-site pairwise comparisons) was correlated with the percentage of agricultural land use or sampling month, and if there was an interaction between these two fixed effects.

We used the R package iNEXT v. 2.0.20 [62] to generate rarefaction and extrapolation curves to estimate the regional diversity in each sampling period (month) for macroinvertebrate families, macroinvertebrate OTUs and chironomid OTUs. To estimate how many undetected taxa remained at each stream site (e.g., locally), we calculated the number of expected total taxa at each based on Chao’s equation using the ‘specpool’ function in the R package vegan [61] and plotted this as the percentage of coverage achieved by dividing the observed number of taxa over the expected number of taxa. Finally, to assess how many rare taxa make up a local community, we calculated the percentage of taxa which only occurred in one (of four) biological replicates at each stream site. We calculated two separate mixed effects models to determine if either the percentage of sampling coverage or the percentage of rare taxa were significantly different between either sampling month or level of identification (i.e., families, all OTUs, chironomid OTUs). All raw data and associated R code are provided as supplementary information (see Additional files 2, 3, 4).

## Results

### Summary and taxonomic richness

We received 74,771,159 sequence reads from the four MiSeq runs (average of 18,893,999 sequences per run), and after post-bioinformatic processing our samples contained a total of 51, 334, 969 reads (average per sample 161,430 ± 69,286 standard deviation) and 2276 OTUs (average 72 per sample ± 33 SD), while all negative controls combined had only 1954 reads (average per control 57 ± 16 SD) and 241 OTUs (average per control 16 ± 13 SD). Notably, our OTU table originally contained 5597 OTUs which were filtered out after clustering for not meeting the 0.01% abundance threshold (these OTUs corresponded to only 33,840 sequences in total). Three OTU sequences were flagged as contaminants through metabaR [59], two of which were unidentified algae and one matched to a species of maple tree (*Acer* sp.*)*, indicating that the most likely cause of the small amount of sequences in the negative controls were tag jumps during sequencing. No samples were flagged as contaminated, and all technical replicates passed our reproducibility criteria. Three samples were removed from the dataset for sequencing depth lower than one standard deviation of the mean (< 86k sequences). The most common reason for an OTU to be removed from the final dataset was a match of less than 90% to the sequence reference database (BOLD). Post metabaR [59], our cleaned dataset contained a total of 41,978,040 high quality reads (177,123 per sample ± 46,881 SD) and 1681 macroinvertebrate OTUs (65 per sample ± 29 SD). We created two more data frames from the cleaned data, one with OTUs scaled back to family-level resolution (145 families total; average of 13 per sample ± 5 SD) and one containing only OTUs from the family Chironomidae (586 OTUs; average of 23 per sample ± 17 SD), as chironomids contained a third of the total diversity within the invertebrate dataset.

**Fig. 2:**
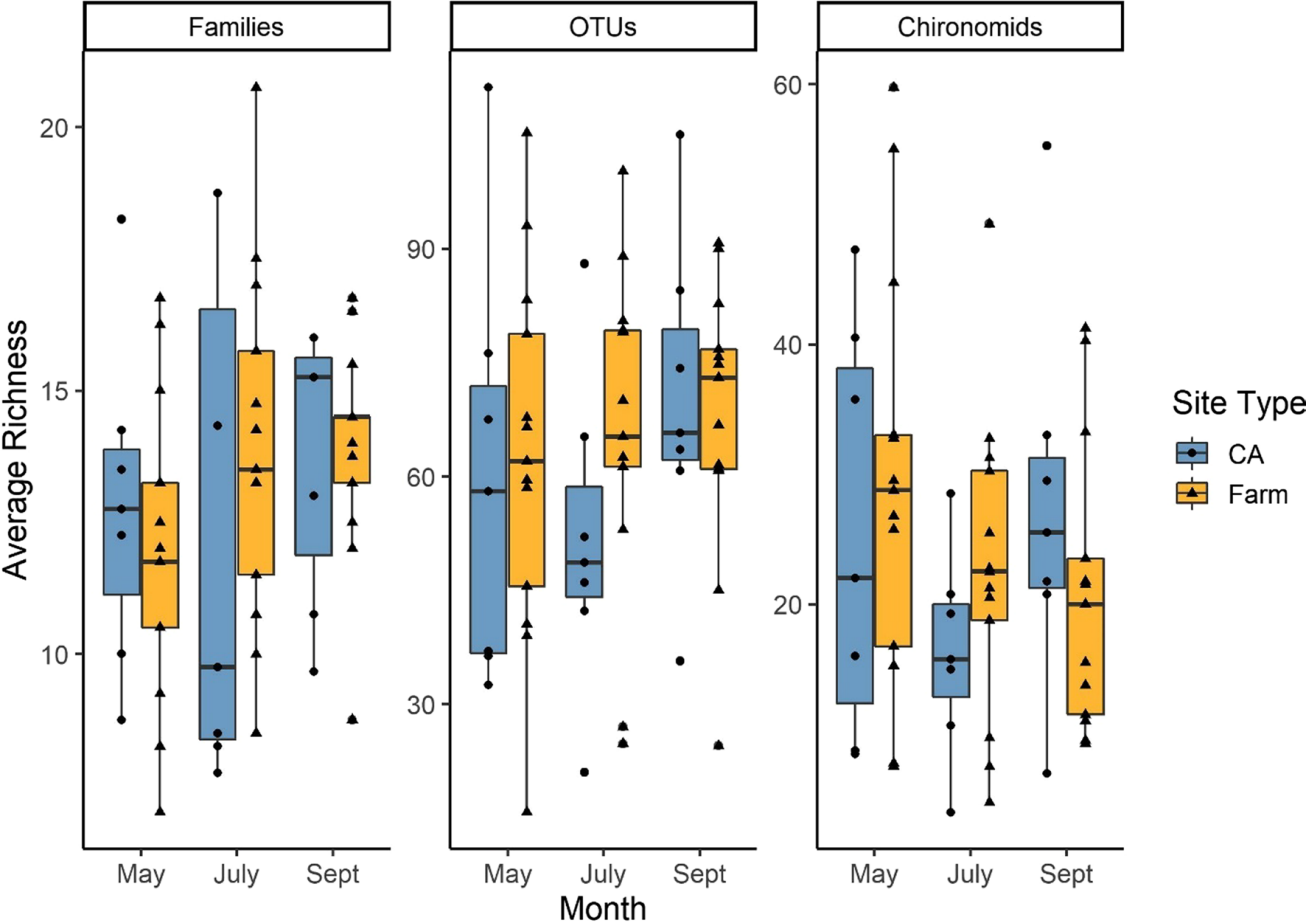
The average taxonomic richness per site in each category (conservation area - CA), n = 7, or farm, n = 13) over the sampling period (May, July, Sept, total n = 237). There was no significant effect of site type or sampling month on taxonomic richness for any group, though sampling month approach significance for chironomid OTUs (F_1,36_ = 3.12, *p* = 0.056). Note the different Y-axis scales in the 3 figures

There was no significant influence of site type (CA versus farm; F_1,18_ = 0.39, *p* = 0.54) or sampling month (F_2, 36_ = 2.62, *p* = 0.09) on average OTU richness (Fig. 2). Likewise, there was no effect of either site type or sampling month on average family richness (type: F_1,18_ = 0.22, *p* = 0.75; month: F_2,36_ = 1.98, *p* = 0.15; Fig. 2). While site type had no effect on average chironomid OTU richness (F_1,18_ = 0.77, *p* = 0.72), there was slight evidence that sampling month was important (though not statistical significant; F_2,36_ = 3.12, *p* = 0.056; Fig. 2). For all above models, there was no significant interaction between site type and sampling month on taxonomic richness.

### Site dissimilarity and agricultural land use

**Fig. 3:**
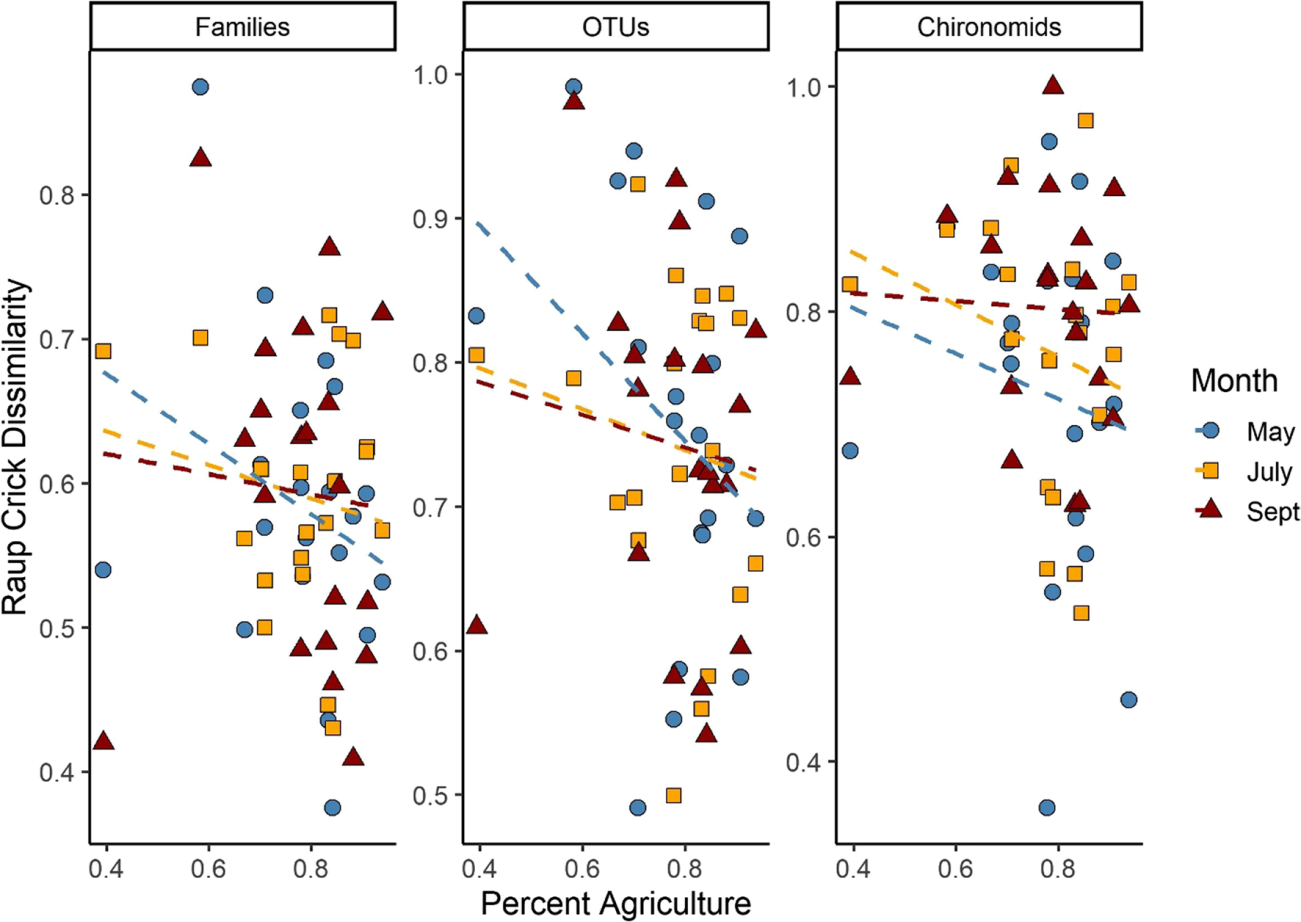
The mean Raup-Crick dissimilarity of all pairwise comparisons within a site is plotted against the percentage of agricultural land use surrounding the stream site. There was no significant relationship between dissimilarity and agriculture or sampling month for any identification level

Mean within-site Raup-Crick dissimilarity was not significantly correlated with the percentage of agricultural land or month for family-level identification (agriculture: F_1,18_ = 1.28, *p* = 0.27; month: F_2,36_ = 0.36, *p* = 0.70), OTU-level identification (agriculture: F_1,18_ = 1.74, *p* = 0.21; month: F_2,36_ = 0.51, *p* = 0.60), or for chironomid OTUs (agriculture: F_1,18_ = 1.26, *p* = 0.27; month: F_1,36_ = 0.09, *p* = 0.91). Dissimilarity was generally high for all sites (i.e., most values within range 0.5–1.0; Fig. 3).

### Rare and undetected taxa

**Fig. 4:**
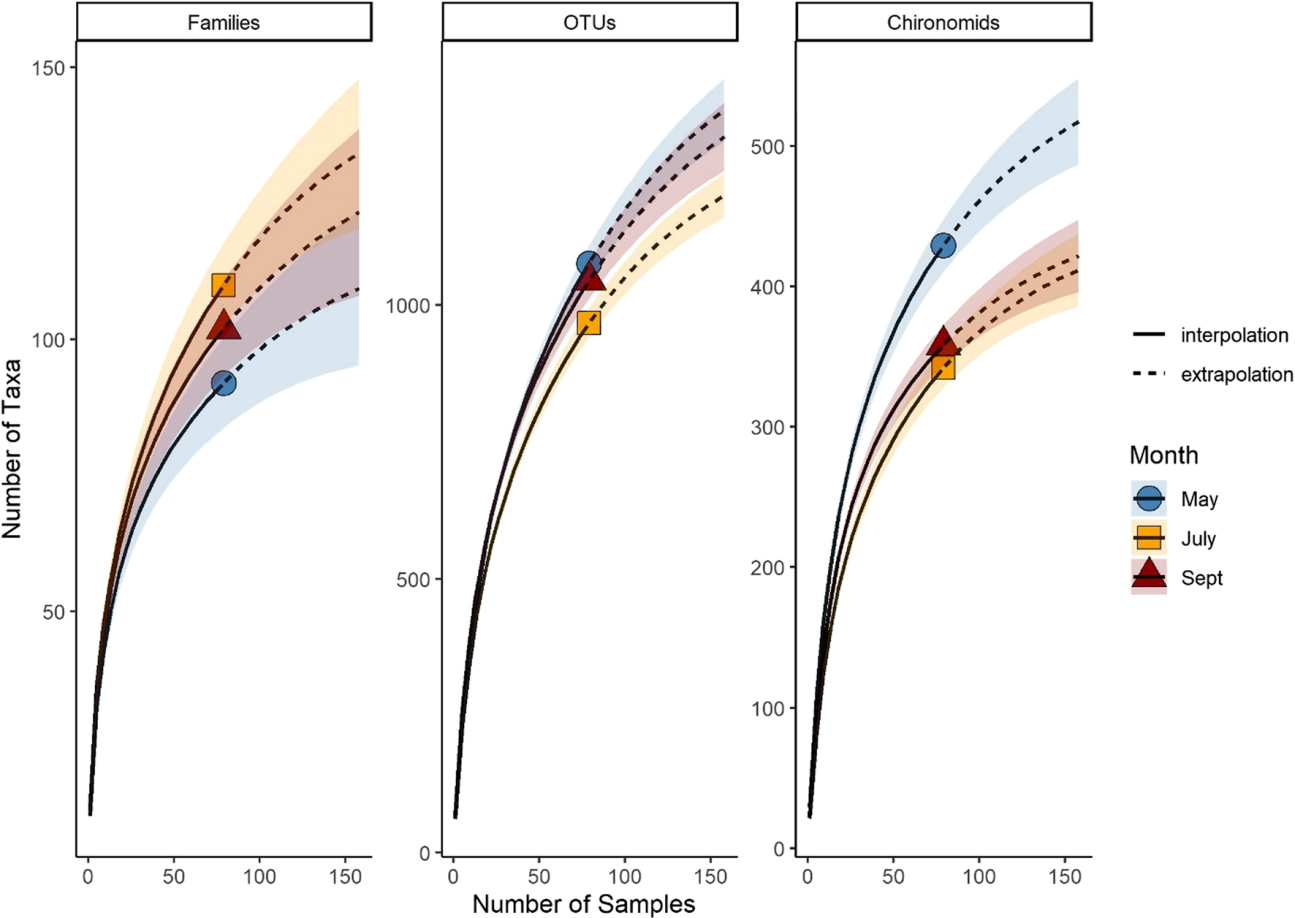
Rarefaction curves for the number of taxa collected each sampling month where the solid line represents interpolated richness from our samples and the dashed line is an extrapolation based on expected number of taxa with continued sampling. Symbols indicate the point where our sampling stopped (n = 80). Note difference in y-axis scales

**Fig. 5:**
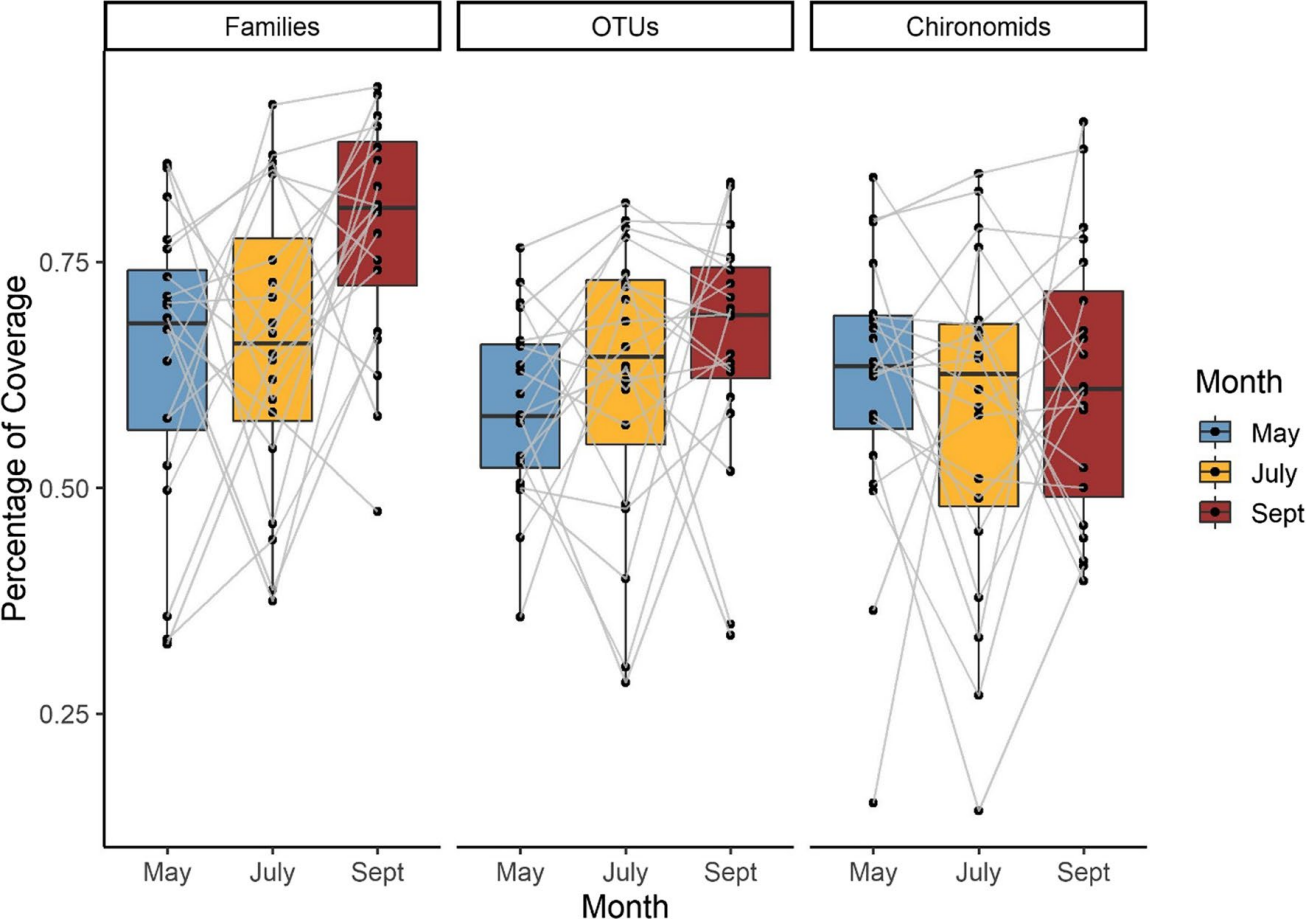
Estimated percentage of taxonomic coverage achieved at each stream by dividing the observed number of taxa over the extrapolated number of taxa expected based on Chao’s equation for each sampling month. Grey lines connect the same stream over time. The amount of ‘undetected’ taxa was significantly different between identification levels (F_2,152_ = 7.5, *p* < 0.001) and sampling months (F_2,152_ = 5.26, *p* < 0.01)

We collected a total of 80 samples for each sampling period, but the species rarefaction curves did not level off (Fig. 4). Extrapolations estimated that at 150 sampling units, the number of new taxa would begin to level off for family-level identification and for chironomid OTUs. Based on Chao’s equation, we determined that each sampling month collected approximately 69% of total invertebrate families present, 62% of all invertebrate OTUs present, and 60% of chironomid OTUs present, and at the stream level this value ranged dramatically from 32–94% for families, 28–84% for all invertebrate OTUs, and 14–90% for chironomid OTUs, indicating that many taxa can be missed locally (Fig. 5). We observed that there were significant effects of both identification level (F_2,152_ = 7.5, *p* < 0.001) and sampling month (F_2,152_ = 5.26, *p* < 0.01). OTU-level resolution (for all invertebrates and just chironomids) had more undetected taxa than family-level resolution and September had the highest coverage for all groups (particularly families).

**Fig. 6:**
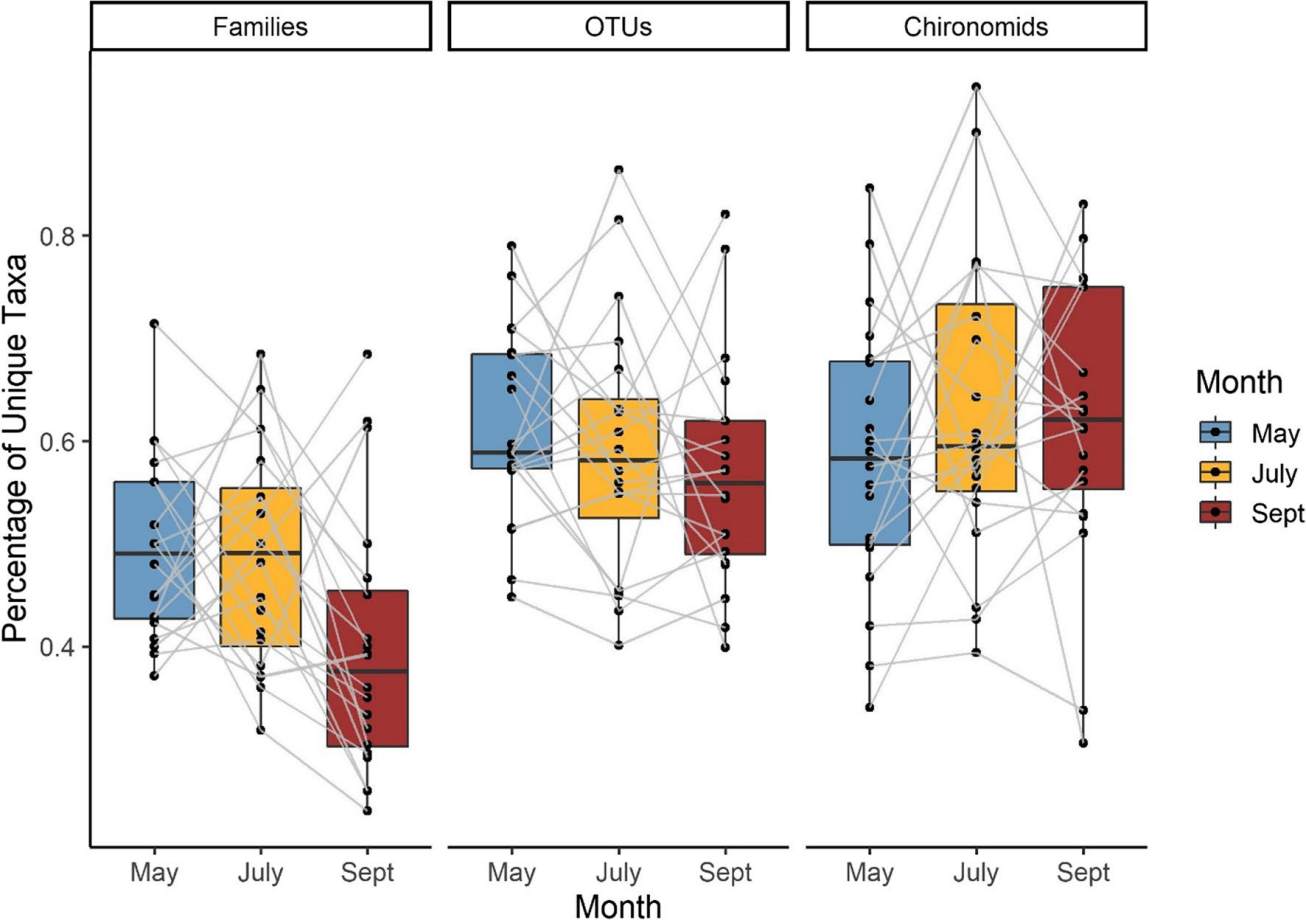
There were four biological replicates at each of the twenty streams to assess small-scale variation in community composition. Bars represent the percentage of the total number of taxa occurring at a stream that were only collected in one of four biological replicates (transects). Grey lines connect the same stream over time. The amount of ‘undetected’ taxa was significantly different between identification levels (F_2,156_ = 37.6, *p* < 0.001). Sampling month had no significant effect on the percentage of rare taxa (F_2,156_ = 2.91, *p* = 0.057)

In addition to undetected taxa, we also calculated the proportion of rare taxa at each stream to determine how variable streams are at small spatial scales. Streams had on average 59% (range 40–87%) of invertebrate OTUs that were only detected in one of four biological replicates, indicating a high degree of turnover within a single site (Fig. 6). Likewise, there was an average of 46% of unique taxa per stream (range 24–71%) for invertebrate families and 61% (range 31–94%) for chironomid OTUs (Fig. 6). There was a significant difference in the number of rare taxa between different identification levels (F_2,156_ = 37.6, *p* < 0.001) and slight evidence that sampling month had an effect (though not statistically significant; F_2,156_ = 2.91, *p* = 0.057). There were less rare taxa in the family-level dataset compared to the OTU and chironomid OTU datasets.

## Discussion

### Rare and missing taxa

Our approach of sampling at small spatial scales within streams revealed incredibly diverse benthic macroinvertebrate communities which varied considerably over short distances. While we collected a total of 80 samples for each sampling month, this was not sufficient to detect biodiversity at the OTU-level at either the local scale (within a stream; alpha diversity) or the regional scale (total species pool; gamma diversity). Even at family-level taxonomic resolution, we consistently underestimated total number of taxa present both locally and regionally, and this increased dramatically for OTUs. Our extrapolations suggest that after 150 samples, the accumulation curve would not be complete. This not only indicates a vast level of diversity masked at the family level, but also suggests that our sampling was not sufficient to capture to complete local community. Doubling our sampling effort (i.e., eight local transects per stream) would be unlikely to represent adequately OTU-level diversity as invertebrate OTUs continued to increase past our extrapolation threshold. While few aquatic metabarcoding papers explore biodiversity at the site level or compare observed versus expected number of taxa, morphological studies using family-level resolution have found similar results as our study. In shallow streams in Brazil, Ligeiro et al. [63] detected 53 invertebrate families and determined that 81% of them were rare (in this case based on a threshold of less than 1% abundance, which differs from our definition). However, Ligeiro et al. [63] concluded that, since sampling a single riffle in a stream collected approximately 75% of invertebrate families present, intensive sampling is not efficient and that the priority should be broad spatial coverage. In contrast, we observed that a single sample within a stream in our study is highly dissimilar in OTU composition from a second point only ten meters away and can represent anywhere between 27 and 85% of the total OTUs detected. Based on our results, we conclude that one sampling point is not sufficient and a more thorough sampling protocol is necessary for a more accurate representation of local diversity. While sampling intensity is an important consideration, a caveat of course is that improvements in sequencing analyses (e.g., increasing read depth) could improve the detection of rare taxa.

Our results more closely resemble terrestrial arthropod metabarcoding studies using malaise traps to explore community composition. In southern Ontario, Steinke et al. [54] surprisingly discovered that ten Malaise traps (tent-like insect collection traps) placed in a row had remarkably high differences in community composition. This field design of trap placement is comparable to our four kicknet transects within a stream, where we similarly found very high within-stream dissimilarity, numerous rare taxa, and large estimates of undetected taxa. For example, Steinke et al. [54] estimated that their OTU pool contained approximately 60% of the total diversity in the region, whereas our estimates were 69% for all invertebrate OTUs (and 78% for chironomid OTUs only). These patterns not only resemble our study in terms of missing taxa, but also display strikingly similar patterns in the number of rare taxa or those occurring only once within a site. Steinke et al. [54] detected close to 3000 OTUs, and almost half of these only occurred once in the same location. At the site level, the percentage of rare taxa in our study (e.g., only occurring at one of four transects in a stream) ranged from 40 to 90% and averaged approximately 60%.

This pattern of arthropod rarity persists across geographic ranges, as a tropical DNA barcoding study using Malaise traps [64] observed very high beta diversity amongst traps, which did not decrease with increased local sampling, indicating that the regional species pool had not been adequately sampled. However, repeated yearly sampling of the same localities decreased beta diversity and allowed D’Souza and Hebert [64] to determine which taxa were present yearly (even if rare) and which were ‘transient’ taxa. D’Souza and Hebert’s [64] study clearly demonstrated the need for both spatially and temporally robust datasets to estimate accurately taxon richness and beta diversity both within a site and over time. In our dataset, we see a large proportion of rare taxa; however, repeated yearly sampling would allow us better power to determine which taxa are rare and which are transient. This is an ongoing challenge of attempting to characterize local communities in streams with no discrete boundaries and of how to delineate local sites in a continuous system. The spatial extent and sampling grain of a study can significantly alter conclusions drawn regarding community composition [65, 66]. It is possible that sampling small spatial scales like in our study can result in an overinflation of local richness due to mass effects swamping out local environmental signal [36], such as taxa being carried to the site due to water flow but not actually being able to establish there (e.g., a “sink” habitat for that species) and thus result in these transient taxa skewing dissimilarity estimates. Alternatively, sampling too large an area can miss changes in community composition in response to environmental signals due to dispersal limitation. Here, we demonstrated a vast amount of both diversity and variability in aquatic macroinvertebrates at both the local and regional scale, underscoring the importance of developing consistent, long-term monitoring programs to assess more accurately patterns in insect declines and thus better inform protection measures.

### Taxonomic richness and land use

While both family and OTU-level richness varied between streams, this was not significantly linked to seasonality or site location (i.e., whether the stream was located on private land or on conservation authority property). This is perhaps unsurprising as local richness (or alpha diversity) can be influenced by a number of habitat parameters in aquatic systems, such as microhabitat availability in streams [28] or hydroperiod in wetlands [67], or can be unrelated to any habitat parameters [63]. While aquatic macroinvertebrates have been successfully used as indicators for decades [14, 68], it is likely that a binary category (e.g., farm or conservation area) was not an effective tool to classify stream quality, especially in a heavily impacted landscape such as southern Ontario. Streams vary in physical habitat parameters, such as the riparian buffer width and the slope of the bank, and these metrics may provide a local buffer from adjacent or upstream landscape-scale agricultural practices [31]. The importance of local habitat conditions has been shown also in previous work in southern Ontario streams by Yates and Bates [69], who determined that aquatic macroinvertebrates (at family-level) were more associated with human activity directly adjacent to the stream such as channel alteration (e.g., decreased sinuosity) and buffer width. In contrast, more mobile fish communities were more responsive to landscape-scale parameters over local conditions in the same system [69]. Fish also have a more evident threshold response in community composition to agricultural impairment, whereas macroinvertebrates (albeit at family-level resolution) respond more gradually to such changes [70] However, there is also the potential that largely developed landscapes, such as southern Ontario, have historically excluded sensitive taxa through centuries of agriculture, and invertebrate communities are already homogenous due to lack of true reference-condition streams [71].

While it is possible these invertebrate communities are homogenous at the family level as suggested by Krynak and Yates [71], and our total family count was generally in concordance with other stream studies in this region using family resolution through morphological identification [69–71], the total OTU richness in our study indicates that vast levels of turnover within a family are possible. At over 1600 OTUs, our metabarcoding richness appears to be much higher than stream metabarcoding studies in various geographic regions [26, 72–74] and more closely resembles richness counts in terrestrial [54, 75] and soil [76] metabarcoding papers. It is likely that bioinformatic decisions in clustering and matching OTUs have a large influence on the taxonomic diversity in a dataset [77]. While our choice of clustering threshold for OTU delineation (97%) is comparable to other studies, it is a more conservative choice in terms of richness than would be achieved at 98% clustering or by using exact or amplicon sequence variants (ESV/ASV) [78] and thus probably not responsible for the high level of diversity found in our dataset. Our OTU count may also be considered a conservative estimate of diversity due to the potential for taxonomic blind spots arising from the use of a single marker (COI) and primer set [79]. We additionally filtered out very low-read OTUs post-clustering (less than 0.01% abundance) which would have reduced the diversity in our dataset. This filtering threshold is commonly used in other invertebrate metabarcoding studies [24, 54], however it decreased the total OTUs in our dataset by half. However, while these OTUs made up 50% of the total OTUs, they only corresponded to 33,840 sequence reads (0.06% of total sequences) and it is important to define a threshold to eliminate OTUs based upon erroneous sequences. We also selected a threshold of 90% sequence similarity to the reference database (BOLD) in order to be included in the final dataset. While many studies use a conservative 98% threshold [26, 54], others have selected a 85% similarity threshold [74]. In our dataset, it is likely that 98% is too strict a cut-off due to the sparsity of reference sequences for understudied taxa such as chironomids. A match of 90% similarity is generally accepted as a ‘family-level’ match in multiple metabarcoding studies [26, 74] and thus aligned well with our approach of comparing OTU and family-level diversity. In addition to bioinformatic sources of bias when estimating total diversity, it is important to note that all stages of a protocol can ultimately alter the number of taxa detected. For example, we incorporated sub-sampling approaches for both removing benthic macroinvertebrates from the bulk sample and taking a representative sample of the subsequent homogenized tissue for DNA extraction. Smaller samples would therefore be more thoroughly sampled than large, dense samples. Laboratory components such as PCR biases can also affect sample diversity as primer biases can result in some taxa being preferentially amplified [48]. The number of PCR cycles will also affect the detection of rare taxa. We encountered a subset of samples which initially resulted in poor sequencing results and subsequently required a higher number of PCR cycles. While this allowed us to include those samples in this manuscript, it does introduce a source of variability when comparing taxa composition.

In our system, chironomids had the highest OTU count of any family. This is consistent with previous aquatic metabarcoding work, notably Beerman et al. [24], who detected nearly 200 chironomid OTUs in a stream mesocosm experiment. Of these total chironomid OTUs, 85% did not have a binomial name assigned from BOLD, which is nearly identical to the value we detected (84.8% without a binomial name in the reference database for high similarity matches). Even with unnamed OTUs, Beerman et al. [24] detected unique responses to environmental stressors (even amongst OTUs which matched to the same binomial name). Reference databases are not complete for many groups, especially very species-rich groups or difficult-to-identify taxa such as aquatic insect larvae, and improvement in species coverages must be made in order to improve the quality of metabarcoding datasets. It is also important to consider that the use of 97% as a clustering threshold can also result in the “splitting” of OTUs (e.g., a single species with large intraspecific divergence of CO1 being split into two OTUs) and thus artificially inflating the OTU count. For example, some chironomid species complexes can have as high as a 10% divergence [80], which would result in multiple OTU clusters when using a 97% threshold. Ultimately, there are numerous bioinformatic decisions which can either increase or decrease the number of OTUs in a metabarcoding study. The vast diversity of the chironomid family in particular merits future study to determine the extent of haplotype diversity which may interfere with establishing discrete OTUs using a set clustering threshold [24, 80].

### Dissimilarity and habitat heterogeneity

Mean Raup-Crick dissimilarity values were consistently high for all levels of taxonomic assignment and sampling season, indicating that our biological replicates were quite unique from each other even when collected at the same stream. While a categorical land use variable did not prove informative in distinguishing macroinvertebrate communities from farm or conservation area streams, we expected the percentage of land used for cropping in the catchment would be a more accurate indication of stream condition. We expected an increased level of agriculture would result in more homogeneous invertebrate communities within a stream (i.e., lower beta diversity) due to the reduction in habitat complexity [34, 35, 38, 39]. We detected no significant relationship between land use and beta diversity [36, 37], and dissimilarity values were quite variable.

Interestingly, we observed steeper declines in dissimilarity values in May along a gradient of agricultural land use for all groups compared to other sampling months, though this trend was not statistically significant. While it is possible this is a spurious correlation, Zizka et al. [81] observed seasonal community changes for aquatic macroinvertebrate OTUs in a study of urban streams. In reference condition (“near natural”) streams, Zizka et al. [81] discovered that the community composition of invertebrates stayed consistent across seasonal sampling periods, whereas communities in more highly impacted streams differed across seasonal sampling periods. This contrasting pattern in seasonal change may be in response to the fact that the inflow of stressors can change over time in impacted areas, resulting in a community-level response that differs over time [81]. It is possible here that changes in water quality or other habitat parameters during the spring resulted in more homogenous communities compared to later in the sampling season. Of course, this may also be due to the emergence of different insect taxa in streams over a season. Our results here indicate that the percentage of agriculture in the landscape is not sufficient here to detect a complicated influence of land use; more detailed parameters are necessary, or our sampling was not sufficient to detect any homogenization, or perhaps the variation in land use in our study was not significant enough to detect a gradient response in community composition.

## Conclusions

Metabarcoding revealed a huge amount of diversity in southern Ontario streams, with many rare or undetected taxa present within each stream. This study highlights the importance of developing robust monitoring plans given the challenges associated with obtaining adequate community composition data for aquatic macroinvertebrate taxa. Field sampling design is an extremely important consideration in monitoring studies, and by using molecular identification we reveal that one sampling point in a stream in not sufficient to detect local diversity. While we detected no discernable response in taxonomic richness or within-stream dissimilarity to agricultural land use, it is possible our interpretation of agriculture land use was too narrow. Given the importance of local parameters to aquatic invertebrate communities, future metabarcoding studies should consider more precise estimates of land use, including both water chemistry and physical parameters, such as stream sinuosity, riparian buffer width, and bank slope to reflect more accurately site condition. In general, our conclusions regarding OTU diversity and rarity were similar to terrestrial metabarcoding work and indicate that aquatic biomonitoring programs can benefit from molecular identification to more accurately reflect trends in insect biodiversity.

## Supplementary Information

**Additional file 1: Appendix 1.** A word document elaborating on data cleaning protocols post-bioinformatic processing using metabaR.

**Additional file 2: Appendix 2.** The R code used to clean the data following the protocol in Appendix 1.

**Additional file 3: Appendix 3.** Raw data post JAMP processing, to be used in Appendix 2. Composed of 4 csv files.

**Additional file 4: Appendix 4.** The R code used to analysis the data and generate figures. This code is meant to run using the output from Appendix 2.

## Data Availability

Raw sequencing data is available at NCBI Sequence Read Archive (Accession: PRJNA783201; https://trace.ncbi.nlm.nih.gov/Traces/sra/sra.cgi?study=SRP347764).

## Abbreviations

ASV: Amplicon sequence variant
BOLD: Barcode of life data system
ESV: Exact sequence variant
OTU: Operational taxonomic unit
